# Supplementary material for: Characterization of Saponins from Various Parts of Platycodon grandiflorum Using UPLC-QToF/MS
Source: Molecules. 2021 Dec 24;27(1):107. doi: 10.3390/molecules27010107 (PMC8746516; doi:10.3390/molecules27010107)
Supplement: Supplementary file 1 [file molecules-27-00107-s001.zip › [Molecules] Supplementary table 2.pdf]

Table S2. Characterization of 38 saponin derivatives in *Platycodon grandiflorum.*

Peak No.	Compounds assignment	RT (min)[1)]	Molecular formula	Theo. Mass $[M+H]^+$ $[M+NH4]^+$	ESI(+)-QToF/MS (experimental ions, m/z) [2)]	
					Exp. Mass $[M+H]^+$ $[M+NH4]^+$	Fragment ions
1	3-*O*-glucosyl-(1→6)-glucosyl-(1→6)-glucosyl-platycodigenin 28-*O*-rhamnosyl-(1→2)-arabinoside (**platycoside G2**)	12.51	$C_{59}H_{96}O_{30}$	1285.5986 1302.6252	1285.6018 1302.6295	1123.55, 1007.50, 961.49, 845.45, 683.39, 521.34
2	3-*O*-glucosyl-(1→6)-glucosyl-(1→6)-glucosyl-platycodigenin 28-*O*-xylosyl-(1→4)-rhamnosyl-(1→2)-arabinoside (**platycoside G1, deapi-platycoside E**)	12.75	$C_{64}H_{104}O_{34}$	1417.6409 1434.6674	1417.6444 1434.6720	1285.60, 1256.59, 1139.54, 1007.50, 977.49, 845.45, 683.39, 667.40, 521.34
3	3-*O*-glucosyl-(1→6)-glucosyl-(1→6)-glucosyl-platycodigenin 28-*O*-apiosyl-(1→3)-xylosyl-(1→4)-rhamnosyl-(1→2)-arabinoside (**platycoside E**)	13.29	$C_{69}H_{112}O_{38}$	1549.6832 1566.7097	1549.6883 1566.7151	1417.64, 1285.60, 1255.59, 1139.54, 1007.50, 845.45, 683.39, 521.34
4	3-*O*-glucosyl-(1→6)-glucosyl-(1→6)-glucosyl-polygalacic acid 28-*O*-apiosyl-(1→3)-xylosyl-(1→4)-rhamnosyl-(1→2)-arabinoside (**platycoside D**)	13.69	$C_{69}H_{112}O_{37}$	1533.6882 1550.7148	1533.6930 1550.7177	1401.65, 1239.59, 1107.55, 961.49, 829.45, 667.40
5 [3)]	3-*O*-glucosyl-(1→6)-glucosyl-(1→6)-glucosyl-platycodigenin 28-*O*-apiosyl-(1→3)-xylosyl-(1→4)-(3"-*O*-acetyl)-rhamnosyl-(1→2)-arabinoside (**3"-*O*-acetyl platycoside E**)	13.72	$C_{71}H_{114}O_{39}$	1591.6937 1608.7203	1591.6962 1608.7233	1459.65, 1255.59, 1139.54, 1007.50, 845.45, 683.39, 521.34
6	3-*O*-glucosyl-(1→3)-glucosyl-platycodigenin 28-*O*-rhamnosyl-(1→2)-arabinoside (**platycoside P**)	14.62	$C_{53}H_{86}O_{25}$	1123.5458 1140.5724	1123.5518 1140.5536	977.49, 845.45, 683.39
7	3-*O*-glucosyl-(1→6)-glucosyl-(1→6)-glucosyl-polygalacic acid 28-*O*-xylosyl-(1→4)-rhamnosyl-(1→2)-arabinoside (**platycoside I, deapi-polygalacin E**)	14.87	$C_{64}H_{104}O_{33}$	1401.6460 1418.6725	1401.6506 1418.6624	1269.60, 1123.55, 991.50, 799.44, 667.40, 637.39, 619.38, 601.37
8	3-*O*-glucosyl-(1→6)-glucosyl-platycodigenin 28-*O*-xylosyl-(1→4)-rhamnosyl-(1→2)-arabinoside (**deapi-platycodin D3**)	15.23	$C_{58}H_{94}O_{29}$	1255.5881 1272.6146	1255.5902 1272.6184	1123.55, 1093.54, 977.49, 961.49, 845.45, 815.44, 683.39, 647.27, 521.34
9 [3)]	3-*O*-glucosyl-(1→6)-glucosyl-(1→6)-glucosyl-platycodigenin 28-*O*-apiosyl-(1→3)-xylosyl-(1→4)-(2"-*O*-acetyl)-rhamnosyl-(1→2)-	15.43	$C_{71}H_{114}O_{39}$	1591.6937 1608.7203	1591.6988 1608.7245	1459.65, 1255.59, 1139.54, 1007.50, 845.45,

	arabinoside (**2"-O-acetyl platycoside E**)					683.39, 521.34
10	3-*O*-glucosyl-(1→6)-glucosyl-platycodigenin 28-*O*-apiosyl-(1→3)-xylosyl-(1→4)-rhamnosyl-(1→2)-arabinoside (**platycodin D3**)	15.75	$C_{63}H_{102}O_{33}$	1387.6303<br>1404.6569	1387.6337<br>1404.6609	1255.59, 1123.55, 1093.53, 977.49, 845.44, 815.44, 683.39, 521.34
11 3)	3-*O*-glucosyl-(1→6)-glucosyl-platycogenic acid A 28-*O*-apiosyl-(1→3)-xylosyl-(1→4)-rhamnosyl-(1→2)-arabinoside (**platyconic acid A3**)	16.05	$C_{63}H_{100}O_{34}$	1401.6096<br>1418.6361	1401.6186<br>1418.6422	1239.5979, 975.48, 945, 813, 667, 535.32
12	3-*O*-glucosyl-(1→6)-glucosyl-platycodigenin 28-*O*-apiosyl-(1→3)-xylosyl-(1→4)-(3"-*O*-acetyl)-rhamnosyl-(1→2)-arabinoside (**3"-O-acetyl platycodin D3**)	16.14	$C_{65}H_{104}O_{34}$	1429.6409<br>1446.6674	1429.6449<br>1446.6723	1297.60, 1165.56, 1003.50, 977.49, 845.45, 815.44, 683.39, 653.38, 503.33
13 3)	3-*O*-glucosyl-(1→6)-glucosyl-platycogenic acid A 28-*O*-apiosyl-(1→3)-xylosyl-(1→4)-(3"-*O*-acetyl)-rhamnosyl-(1→2)-arabinoside (**3"-O-acetyl platyconic acid A3**)	16.43	$C_{65}H_{102}O_{35}$	1443.6202<br>1460.6467	1443.6250<br>1460.6512	1311.58, 1017.48, 829.45, 667.40, 535.32
14	3-*O*-glucosyl-(1→6)-glucosyl-polygalacic acid 28-*O*-apiosyl-(1→3)-xylosyl-(1→4)-rhamnosyl-(1→2)-arabinoside (**polygalacin D3**)	16.67	$C_{63}H_{102}O_{32}$	1371.6354<br>1388.6620	1371.6394<br>1388.6667	1239.59, 1107.55, 961.49, 829.45, 799.44, 783.45, 667.40, 619.38, 487.34
15	3-*O*-glucosyl-(1→6)-glucosyl-platycodigenin 28-*O*-xylosyl-(1→4)-(2"-*O*-acetyl)-rhamnosyl-(1→2)-arabinoside (**deapi-2"-O-acetyl platycodin D3**)	16.99	$C_{60}H_{96}O_{30}$	1297.5986<br>1314.6252	1297.6021<br>1314.6292	1165.56, 1135.55, 977.49, 845.45, 815.44, 683.39, 521.34
16	3-*O*-glucosyl-(1→6)-glucosyl-polygalacic acid 28-*O*-apiosyl-(1→3)-xylosyl-(1→4)-(3"-*O*-acetyl)-rhamnosyl-(1→2)-arabinoside (**3"-O-acetyl polygalacin D3**)	17.09	$C_{65}H_{104}O_{33}$	1413.6460<br>1430.6725	1413.6503<br>1430.6754	1281.60, 1149.56, 961.49, 829.45, 825.46, 667.40, 505.35
17	3-*O*-glucosyl-(1→6)-glucosyl-platycodigenin 28-*O*-apiosyl-(1→3)-xylosyl-(1→4)-(2"-*O*-acetyl)-rhamnosyl-(1→2)-arabinoside (**2"-O-acetyl platycodin D3**)	17.64	$C_{65}H_{104}O_{34}$	1429.6409<br>1446.6674	1429.6447<br>1446.6723	1297.60, 1165.56, 1135.55, 1033.51, 1003.50, 977.49, 845.45, 815.44, 683.39
18 3)	3-*O*-glucosyl-(1→6)-glucosyl-platycogenic acid A 28-*O*-apiosyl-(1→3)-xylosyl-(1→4)-(2"-*O*-acetyl)-rhamnosyl-(1→2)-arabinoside (**2"-O-acetyl platyconic acid A3**)	18.02	$C_{65}H_{102}O_{35}$	1443.6202<br>1460.6467	1443.6442<br>1460.6518	1311.58, 1017.48, 829.45, 667.40, 535.32
19	3-*O*-glucosyl-(1→3)-glucosyl-platycodigenin 28-*O*-xylosyl-(1→4)-rhamnosyl-(1→2)-arabinoside (**deapi-platycodin D2, platycoside**	18.43	$C_{58}H_{94}O_{29}$	1255.5881<br>1272.6146	1255.5912<br>1272.6184	1123.55, 1093.53, 977.49, 961.49, 845.45, 815.44, 799.44, 683.39, 521.34

	A)					
20 3)	3-*O*-glucuronosyl-platycodigenin 28-*O*-apiosyl-(1→3)-xylosyl-(1→4)-rhamnosyl-(1→2)-arabinoside (**platycurodin D**)	18.49	$C_{57}H_{90}O_{29}$	1239.5568<br>1256.5833	1239.5616<br>1256.5949	1107.51, 975.47, 829.42, 811.40, 697.37, 679.36, 521.34, 485.32
21	3-*O*-glucosyl-(1→6)-glucosyl-polygalacic acid 28-*O*-apiosyl-(1→3)-xylosyl-(1→4)-(2''-*O*-acetyl)-rhamnosyl-(1→2)-arabinoside (**2''-O-acetyl polygalacin D3**)	18.58	$C_{65}H_{104}O_{33}$	1413.6460<br>1430.6725	1413.6501<br>1430.6718	1239.56, 1149.56, 1107.51, 961.49, 829.45, 799.44, 667.40, 487.34
22	3-*O*-glucosyl-(1→3)-glucosyl-platycodigenin 28-*O*-xylosyl-(1→4)-(3''-*O*-acetyl)-rhamnosyl-(1→2)-arabinoside (**deapi-3''-O-acetyl platycodin D2**)	18.80	$C_{60}H_{96}O_{30}$	1297.5986<br>1314.6252	1297.6010<br>1314.6292	1165.56, 1135.55, 977.49, 845.45, 815.44, 683.39, 521.34
23	3-*O*-glucuronosyl-platycodigenin 28-*O*-apiosyl-(1→3)-xylosyl-(1→4)-(3''-*O*-acetyl)-rhamnosyl-(1→2)-arabinoside (**3''-O-acetyl platycurodin D, platycodin L**)	18.87	$C_{59}H_{92}O_{30}$	1281.5673<br>1298.5939	1281.5724<br>1298.6042	1149.52, 1017.48, 999.47, 841.45, 829.42, 697.37, 679.36, 653.38, 521.34
24	3-*O*-glucosyl-(1→3)-glucosyl-platycodigenin 28-*O*-apiosyl-(1→3)-xylosyl-(1→4)-rhamnosyl-(1→2)-arabinoside (**platycodin D2**)	18.97	$C_{63}H_{102}O_{33}$	1387.6303<br>1404.6569	1387.6344<br>1404.6610	1255.59, 1123.55, 1093.53, 977.49, 961.49, 845.45, 815.44, 683.39, 521.34
25	3-*O*-glucosyl-platycodigenin 28-*O*-apiosyl-(1→3)-xylosyl-(1→4)-rhamnosyl-(1→2)-arabinoside (**platycodin D**)	19.08	$C_{57}H_{92}O_{28}$	1225.5775<br>1242.6041	1225.5809<br>1242.6084	1093.53, 1063.52, 961.49, 931.48, 845.45, 815.44, 799.44, 683.39, 521.34
26	3-*O*-glucosyl-(1→3)-glucosyl-platycodigenin 28-*O*-apiosyl-(1→3)-xylosyl-(1→4)-(3''-*O*-acetyl)-rhamnosyl-(1→2)-arabinoside (**3''-O-acetyl platycodin D2**)	19.28	$C_{65}H_{104}O_{34}$	1429.6409<br>1446.6674	1429.6440<br>1446.6723	1297.60, 1165.56, 1135.55, 1033.50, 1003.50, 977.49, 845.45, 815.44, 683.39
27	3-*O*-glucosyl-platycodigenin 28-*O*-apiosyl-(1→3)-xylosyl-(1→4)-(3''-*O*-acetyl)-rhamnosyl-(1→2)-arabinoside (**3''-O-acetyl platycodin D, platycodin C**)	19.44	$C_{59}H_{94}O_{29}$	1267.5881<br>1284.6146	1267.5919<br>1284.6187	1135.54, 1105.53, 1003.50, 973.49, 841.45, 815.44, 683.39, 653.38, 521.34
28	3-*O*-glucosyl-(1→3)-glucosyl-polygalacic acid 28-*O*-apiosyl-(1→3)-xylosyl-(1→4)-(3''-*O*-acetyl)-rhamnosyl-(1→2)-arabinoside (**3''-O-acetyl polygalacin D2**)	19.59	$C_{65}H_{104}O_{33}$	1413.6460<br>1430.6725	1413.6500<br>1430.6760	1281.60, 1209.58, 1149.56, 1089.54, 987.51, 807.45, 783.45, 637.39, 487.34
29	3-*O*-glucosyl-platycogenic acid A 28-*O*-xylosyl-(1→4)-(2''-*O*-acetyl)-rhamnosyl-(1→2)-arabinoside (**2''-O-acetyl platyconic acid C,**	19.76	$C_{54}H_{84}O_{26}$	1149.5251<br>1165.5516	1149.5309<br>1165.5587	1089.54, 1017.48, 999.51, 957.50, 829.42, 667.40

	**platyconic acid D**)					
30	3-*O*-glucosyl-polygalacic acid 28-*O*-apiosyl-(1→3)-xylosyl-(1→4)-(3''-*O*-acetyl)-rhamnosyl-(1→2)-arabinoside (**3''-O-acetyl polygalacin D**)	19.95	$C_{59}H_{94}O_{28}$	1251.5932<br>1268.6197	1251.5976<br>1268.6234	1119.55, 987.51, 825.46, 799.44, 649.35, 505.35, 487.34, 469.32
31	3-*O*-glucosyl-platycogenic acid A 28-*O*-apiosyl-(1→3)-xylosyl-(1→4)-rhamnosyl-(1→2)-arabinoside (**platyconic acid A**)	19.95	$C_{57}H_{90}O_{29}$	1239.5568<br>1256.5833	1239.5630<br>1256.5874	1107.51, 975.47, 829.42, 813.42, 697.37, 667.37, 649.35, 535.32
32	3-*O*-glucosyl-platycogenic acid A 28-*O*-apiosyl-(1→3)-xylosyl-(1→4)-(3''-*O*-acetyl)-rhamnosyl-(1→2)-arabinoside (**3''-O-acetyl platyconic acid A**)	20.17	$C_{59}H_{92}O_{30}$	1281.5673<br>1298.5939	1281.5731<br>1298.5988	1149.52, 1017.48, 987.51, 855.47, 829.42, 697.37, 667.36, 535.32
33	3-*O*-glucuronosyl-platycodigenin 28-*O*-apiosyl-(1→3)-xylosyl-(1→4)-(2''-*O*-acetyl)-rhamnosyl-(1→2)-arabinoside (**2''-O-acetyl platycurodin D, platycodin K**)	20.33	$C_{59}H_{92}O_{30}$	1281.5673<br>1298.5939	1281.5749<br>1298.5988	1149.53, 1131.51, 1017.48, 999.47, 829.42, 697.37, 679.36, 521.34
34	3-*O*-glucosyl-(1→3)-glucosyl-platycodigenin 28-*O*-apiosyl-(1→3)-xylosyl-(1→4)-(2''-*O*-acetyl)-rhamnosyl-(1→2)-arabinoside (**2''-O-acetyl platycodin D2, platycodin V**)	20.58	$C_{65}H_{104}O_{34}$	1429.6409<br>1446.6674	1429.6428<br>1446.6713	1297.60, 1267.58, 1165.56, 1003.50, 977.49, 845.45, 683.39, 653.38, 521.34
35	3-*O*-glucosyl-platycodigenin 28-*O*-apiosyl-(1→3)-xylosyl-(1→4)-(2''-*O*-acetyl)-rhamnosyl-(1→2)-arabinoside (**2''-O-acetyl platycodin D, platycodin A**)	20.81	$C_{59}H_{94}O_{29}$	1267.5881<br>1284.6146	1267.5909<br>1284.6199	1135.54, 1105.54, 1003.50, 973.49, 841.45, 815.44, 683.39, 653.38, 521.34
36	3-*O*-glucosyl-(1→3)-glucosyl-polygalacic acid 28-*O*-apiosyl-(1→3)-xylosyl-(1→4)-(2''-*O*-acetyl)-rhamnosyl-(1→2)-arabinoside (**2''-O-acetyl polygalacin D2**)	20.95	$C_{65}H_{104}O_{33}$	1413.6460<br>1430.6725	1413.6492<br>1430.6755	1281.60, 1149.55, 1089.54, 987.51, 961.49, 829.45, 799.45, 667.40, 505.35
37	3-*O*-glucosyl-polygalacic acid 28-*O*-apiosyl-(1→3)-xylosyl-(1→4)-(2''-*O*-acetyl)-rhamnosyl-(1→2)-arabinoside (**2''-O-acetyl polygalacin D**)	21.38	$C_{59}H_{94}O_{28}$	1251.5932<br>1268.6197	1251.5966<br>1268.6233	1119.55, 1089.54, 987.51, 825.46, 799.44, 667.40, 505.35
38 [3)]	3-*O*-glucosyl-platycogenic acid A 28-*O*-apiosyl-(1→3)-xylosyl-(1→4)-(2''-*O*-acetyl)-rhamnosyl-(1→2)-arabinoside (**2''-O-acetyl platyconic acid A**)	21.65	$C_{59}H_{92}O_{30}$	1281.5673<br>1298.5939	1281.5751<br>1298.5995	1149.52, 1017.48, 999.47, 855.43, 829.42, 697.37, 535.32

[1)]RT : The roots (containing peel) of Platycodi Radix represents RT (retention time), except for peak 6 reffered by the blanched roots (without peel) of Platycodi Radix.
[2)]All samples are analyzed in positive ionization mode. [3)]New compounds are identified.
