# Supplementary material for: Characterization of Saponins from Various Parts of Platycodon grandiflorum Using UPLC-QToF/MS
Source: Molecules. 2021 Dec 24;27(1):107. doi: 10.3390/molecules27010107 (PMC8746516; doi:10.3390/molecules27010107)
Supplement: Supplementary file 1 [file molecules-27-00107-s001.zip › [Molecules] Supplementary table 1.pdf]

Table S1. Characterization of 38 saponin derivatives in *Platycodon grandiflorum.*

No.	Compound names	MW[1)]	Fragment ions pattern		Used parts	Literature cited[4)]
			Positive[2)]	Negative[3)]		
1	**16-oxo-2β,3β,23,24-tetrahydroxy-olean-12-ene** (platycodonoid A)	474	**497**, 475, 457, 439, 421, 409		Roots	35
2	3-*O*-glucosyl-**platycodonoid A** (platycodonoid B)	636	**659**, 637, 475, 457, 439, 421		Roots	35
3	3-*O*-glucosyl-**polygalacic acid**	666	**689**, 667, 505, 487, 469		Roots	7, 35
4	3-*O*-glucosyl-**platycogenic acid A** lactone (platycoside M-1)	678	**701**, 679, 517, 499		Roots	9
5	3-*O*-glucosyl-**platycodigenin**	682	705, 683, 521, 503, 485, 467	**681**	Roots	1, 32, 35
6	3-*O*-glucosyl-**2β,12α,16α,23,24-pentahydroxy-oleanane-28(13)-lactone**	698	**721**, 699, 537, 519, 501		Roots	36
7	3-*O*-glucosyl-**2β,3β,16β,21β-tetrahydroxy-olean-12-ene-28-oic acid** 16-*O*-xyloside (platycodon B)	798	821, 799, 667, 637, 505, 487, 469	**797, 635, 503**	Roots	25
8	3-*O*-glucosyl-**2β,3β,16β,21β-tetrahydroxy-olean-12-ene-28-oic acid** 16-*O*-glucoside (platycodon A)	828	851, 829, 667, 505, 487, 469	**827, 665, 503**	Roots	25
9	2-*O*-glucosyl-**2β,3β,16α,21β-tetrahydroxy-olean-12-ene-28-oic acid** 21-*O*-glucoside (platycosaponin A)	828	851, **829**, 667, 505, 487, 469		Roots	32
10	3-*O*-glucosyl-(1→6)-glucosyl-**polygalacic acid** (3-*O*-gentiobiosylpolygalacic acid)	828	**851**, 829, 667, 505, 487, 469	**827, 665**	Roots	26
11	3-*O*-glucosyl-(1→3)-glucosyl-**polygalacic acid** (3-*O*-laminaribiosylpolygalacic acid)	828	**851**, 829, 667, 505, 487, 469		Roots	7
12	3-*O*-glucosyl-(1→6)-glucosyl-**platycodigenin** (3-*O*-gentiobiosylplatycodigenin) (platycoside L)	844	**867, 845**, 683, 521, 503, 485, 467		Roots	8, 38
13	3-*O*-glucosyl-(1→3)-glucosyl-**platycodigenin** (3-*O*-laminaribiosylplatycodigenin;platycoside K)	844	**867, 845**, 683, 521, 503, 485, 467		Roots	8
14	3-*O*-glucosyl-(1→3)-glucosyl-**2β,12α,16α,23α-tetrahydroxy-oleanane-28(13)-lactone**	844	**867**, 845, 683, 521, 503	**843, 681, 519**	Roots	36
15	3-*O*-glucosyl-**platycogenic acid A** lactone 28-*O*-rhamnosyl-(1→2)-arabinoside	956	**979**, 957, 811,		Roots	9

	(platycoside M-2)		795, 679, 649, 517, 499			
16	3-*O*-glucosyl-**platycodigenin** 28-*O*-rhamnosyl-(1→2)-arabinoside (platycoside F)	960	983, 961, 815, 799, 683, 653, 521, 503, 485, 467		Roots	6, 8, 32
17	3-*O*-glucosyl-(1→6)-glucosyl-(1→6)-glucosyl-**platycodigenin** (3-*O*-gentiotriosylplatycodigenin)	1006	**1029, 1007**, 845, 683, 521, 503, 485, 467	**1005, 843, 681, 519**	Roots	26, 38
18	3-*O*-glucosyl-**polygalacic acid** 28-*O*-xylosyl-(1→4)-rhamnosyl-(1→2)-arabinoside (deapi-polygalacin D;platycoside J)	1076	**1099**, 1077, 945, 915, 799, 783, 667, 637, 505, 487, 469		Roots	6, 8
19	3-*O*-glucosyl-**platycogenic acid A** lactone 28-*O*-xylosyl-(1→4)-rhamnosyl-(1→2)-arabinoside (platycoside M-3)	1088	**1111**, 1089, 957, 927, 811, 795, 679, 649, 517, 499		Roots	6, 9
20	3-*O*-glucosyl-**platycodigenin** 28-*O*-xylosyl-(1→4)-rhamnosyl-(1→2)-arabinoside (deapi-platycodin D)	1092	**1115**, 1093, 961, 931, 815, 799, 683, 653, 521, 503, 485, 467	**1091, 959, 681, 663, 519**	Roots	4, 5, 10, 11, 12, 13, 16, 17, 18, 20, 21, 23, 26, 32, 35, 38
21	3-*O*-glucosyl-**platycogenic acid A** 28-*O*-xylosyl-(1→4)-rhamnosyl-(1→2)-arabinoside (platyconic acid C)	1106	1129, **1107**, 975, 945, 829, 813, 697, 667, 535, 517, 499		Roots	32
22	3-*O*-glucosyl-(1→6)-glucosyl-**polygalacic acid** 28-*O*-rhamnosyl-(1→2)-arabinoside (platycoside N)	1106	1129, 1107, 961, 945, 829, 799, 783, 667, 637, 505, 487, 469		Roots	23
23	3-*O*-glucosyl-(1→6)-glucosyl-**platycogenic acid A** lactone 28-*O*-rhamnosyl-(1→2)-arabinoside (platycoside Q)	1118	1141, 1119, 973, 957, 841, 811, 795, 679, 649, 517, 499	**1117**	Roots	29
24	3-*O*-glucosyl-**polygalacic acid** 28-*O*-xylosyl-(1→4)-(3''-*O*-acetyl)rhamnosyl- (1→2)-arabinoside (deapi-3''-*O*-acetylpolygalacin D)	1118	1141, 1119, 987, 957, 825, 799, 667, 637, 505, 487, 469		Roots	18
25	3-*O*-glucosyl-24*β*-methyl-**platycogenic acid A** 28-*O*-xylosyl-(1→4)-rhamnosyl-(1→2)-arabinoside (platycoside O)	1120	**1143**, 1121, 989, 959, 843, 827,		Roots	6

			711, 681, 549, 531, 513			
26	3-*O*-glucosyl-(1→3)-glucosyl-**platycodigenin** 28-*O*-rhamnosyl-(1→2)-arabinoside (platycoside P)	1122	1145, 1123, 977, 961, 845, 815, 799, 683, 653, 521, 503, 485, 467	**1121**	Roots	29
27	3-*O*-glucosyl-**platycodigenin** 28-*O*-xylosyl-(1→4)-(2''-*O*-acetyl)rhamnosyl- (1→2)-arabinoside (deapi-2''-*O*-acetylplatycodin D; platycoside B)	1134	1157, **1135**, 1003, 973, 841, 815, 683, 653, 521, 503, 485, 467	1133	Roots	6, 8, 16, 32, 38
28	3-*O*-glucosyl-**platycodigenin** 28-*O*-xylosyl-(1→4)-(3''-*O*-acetyl)rhamnosyl- (1→2)-arabinoside (deapi-3''-*O*-acetylplatycodin D; platycoside C)	1134	1157, **1135**, 1003, 973, 841, 815, 683, 653, 521, 503, 485, 467	**1133, 1091, 1001, 681, 663, 619**	Roots	8, 16, 17, 24, 38
29	3-*O*-glucosyl-**platycogenic acid A** 28-*O*-xylosyl-(1→4)-(2''-*O*-acetyl)rhamnosyl-(1→2)-arabinoside (2''-*O*-acetylplatyconic acid C; platyconic acid D)	1148	1171, **1149**, 1017, 987, 855, 829, 697, 667, 535, 517, 499		Roots	32
30	3-*O*-glucosyl-(1→6)-glucosyl-**polygalacic acid** 28-*O*-(2''-*O*-acetyl)rhamnosyl-(1→2)-arabinoside (dexyl-2''-*O*-acetylpolygalacin D_3)	1148	1171, **1149**, 987, 961, 829, 825, 799, 667, 637, 505, 487, 469	**1147**	Roots	16, 17, 38
31	3-*O*-glucosyl-(1→6)-glucosyl-**polygalacic acid** 28-*O*-(3''-*O*-acetyl)rhamnosyl-(1→2)-arabinoside (dexyl-3''-*O*-acetylpolygalacin D_3)	1148	1171, **1149**, 987, 961, 829, 825, 799, 667, 637, 505, 487, 469	**1147**	Roots	16, 38
32	3-*O*-glucosyl-**polygalacic acid** 28-*O*-apiosyl-(1→3)-xylosyl-(1→4)-rhamnosyl-(1→2)-arabinoside (polygalacin D)	1208	**1231**, 1209, 1077, 1047, 945, 915, 799, 783, 667, 637, 505, 487, 469	**1207, 1075, 943, 665**	Roots	2, 4, 5, 7, 10, 12, 13, 15, 16, 18, 19, 21, 24, 34, 35, 37
33	3-*O*-glucosyl-16-oxo-**platycodigenin** 28-*O*-apiosyl-(1→3)-xylosyl-(1→4)-rhamnosyl-(1→2)-arabinoside (16-oxo-platycodin D)	1222	**1245, 1223**, 1091, 1061, 959, 929, 813, 797, 681, 651, 519, 501, 483, 465		Roots	22
34	3-*O*-glucosyl-**platycodigenin** 28-*O*-apiosyl-(1→3)-xylosyl-(1→4)-rhamnosyl-(1→2)-arabinoside	1224	**1247, 1225, 1093**, 1063, **961, 931,**	**1223, 1205, 1091, 1073,**	Leaves, Stems,	2, 3, 10, 11, 12, 13, 15, 16, 17, 18, 19, 20, 21,

	(platycodin D)		**815, 799, 683**, 653, 521, 503, 485, 467	**959, 681, 519**	Roots	22, 23, 26, 28, 30, 31, 32, 34, 35, 37, 38
35	3-*O*-glucosyl-**platycogenic acid A** 28-*O*-apiosyl-(1→3)-xylosyl-(1→4)-rhamnosyl-(1→2)-arabinoside (platyconic acid A)	1238	1261, 1239, 1107, 1077, 975, 945, 829, 813, 697, 667, 535, 517, 499		Roots	2, 3, 18, 19, 34
36	3-*O*-glucosyl-(1→3)-glucosyl-**polygalacic acid** 28-*O*-xylosyl-(1→4)-rhamnosyl-(1→2)-arabinoside (deapi-polygalacin D_2)	1238	1261, 1239, 1107, 1077, 961, 945, 915, 829, 799, 783, 667, 637, 505, 487, 469	**1237**	Roots	17
37	3-*O*-glucosyl-(1→6)-glucosyl-**polygalacic acid** 28-*O*-xylosyl-(1→4)-rhamnosyl-(1→2)-arabinoside (deapi-polygalacin D_3; platycoside H)	1238	**1261**, 1239, 1107, 1077, 961, 945, 915, 829, 799, 783, 667, 637, 505, 487, 469	**1237, 1105, 827, 809, 665, 647**	Roots	8, 16, 26
38	3-*O*-glucosyl-(1→6)-glucosyl-**platycogenic acid A** lactone 28-*O*-xylosyl-(1→4)-rhamnosyl-(1→2)-arabinoside (deapi-platyconic acid B lactone)	1250	**1273**, 1251, 1119, 1089, 973, 957, 927, 841, 811, 795, 679, 649, 517, 499		Roots	2
39	3-*O*-glucosyl-**polygalacic acid** 28-*O*-apiosyl-(1→3)-xylosyl-(1→4)-(2''-*O*-acetyl)rhamnosyl-(1→2)-arabinoside (2''-*O*-acetylpolygalacin D)	1250	1273, 1251, 1119, 1089, 987, 957, 825, 799, 667, 637, 505, 487, 469	**1249, 1207, 1117, 985, 665**	Roots	2, 10, 12, 13, 15, 16, 24, 32, 34, 37
40	3-*O*-glucosyl-**polygalacic acid** 28-*O*-apiosyl-(1→3)-xylosyl-(1→4)-(3''-*O*-acetyl)rhamnosyl-(1→2)-arabinoside (3''-*O*-acetylpolygalacin D)	1250	1273, 1251, 1119, 1089, 987, 957, 825, 799, 667, 637, 505, 487, 469	**1249, 1207, 1117, 985, 665**	Roots	2, 10, 12, 13, 15, 18, 19, 24
41	3-*O*-glucosyl-(1→3)-glucosyl-**platycodigenin** 28-*O*-xylosyl-(1→4)-rhamnosyl-(1→2)-arabinoside (deapi-platycodin D_2; platycoside A)	1254	**1277, 1255**, 1123, 1093, 977, 961, 931, 845, 815, 799, 683, 653, 521, 503, 485, 467	**1253, 843**	Leaves, Stems, Roots	2, 20, 24, 31, 32
42	3-*O*-glucosyl-(1→6)-glucosyl-**platycodigenin** 28-*O*-xylosyl-(1→4)-rhamnosyl-(1→2)-arabinoside (deapi-platycodin D_3)	1254	1277, **1255**, 1123, 1093, 977, 961,	**1253, 1121, 1091, 843**	Roots	4, 11, 12, 13, 16, 17, 18, 23,

			931, 845, 815, 799, 683, 653, 521, 503, 485, 467			24, 32, 34, 38
43	3-*O*-glucosyl-**platycodigenin** 28-*O*-apiosyl-(1→3)-xylosyl-(1→4)-(2''-*O*-acetyl)rhamnosyl-(1→2)-arabinoside (2''-*O*-acetylplatycodin D; platycodin A)	1256	**1289, 1267**, 1135, 1105, 1003, 973, 841, 815, 683, 653, 521, 503, 485, 467	**1265, 1223, 1133, 1001, 795, 681**	Leaves, Stems, Roots	2, 10, 12, 13, 15, 16, 17, 18, 19, 20, 24, 32, 34, 37, 38
44	3-*O*-glucosyl-**platycodigenin** 28-*O*-apiosyl-(1→3)-xylosyl-(1→4)-(3''-*O*-acetyl)rhamnosyl-(1→2)-arabinoside (3''-*O*-acetylplatycodin D; platycodin C)	1266	**1289, 1267**, 1135, 1105, 1003, 973, 841, 815, 683, 653, 521, 503, 485, 467	**1265, 1223, 1133, 795, 681**	Roots	2, 10, 15, 16, 17, 18, 19, 24, 34, 38
45	3-*O*-glucosyl-**platycogenic acid A** 28-*O*-apiosyl-(1→3)-xylosyl-(1→4)-(3''-*O*-acetyl)rhamnosyl-(1→2)-arabinoside (3''-*O*-acetylplatyconic acid A)	1280	1303, 1281, 1149, 1119, 1017, 987, 855, 829, 697, 667, 535, 517, 499		Roots	34
46	3-*O*-glucuronyl-**platycodigenin** 28-*O*-apiosyl-(1→3)-xylosyl-(1→4)-(2''-*O*-acetyl)rhamnosyl-(1→2)-arabinoside (2''-*O*-acetyl platycurodin D; platycodin K)	1280	1303, **1281**, 1149, 1105, 1017, 973, 841, 829, 697, 653, 521, 503, 485, 467		Roots	32
47	3-*O*-glucuronyl-**platycodigenin** 28-*O*-apiosyl-(1→3)-xylosyl-(1→4)-(3''-*O*-acetyl)rhamnosyl-(1→2)-arabinoside (3''-*O*-acetyl platycurodin D; platycodin L)	1280	1303, **1281**, 1149, 1105, 1017, 973, 841, 829, 697, 653, 521, 503, 485, 467		Roots	32
48	3-*O*-glucosyl-(1→3)-glucosyl-**polygalacic acid** 28-*O*-xylosyl-(1→4)-(2''-*O*-acetyl)rhamnosyl- (1→2)-arabinoside (deapi-2''-*O*-acetylpolygalacin D_2)	1280	1303, **1281**, 1149, 1119, 987, 961, 957, 829, 825, 825, 799, 667, 637, 505, 487, 469	**1279**	Roots	16, 17, 38
49	3-*O*-glucosyl-(1→3)-glucosyl-**polygalacic acid** 28-*O*-xylosyl-(1→4)-(3''-*O*-acetyl)rhamnosyl- (1→2)-arabinoside (deapi-3''-*O*-acetylpolygalacin D_2)	1280	1303, **1281**, 1149, 1119, 987, 961, 957, 829, 825, 825, 799, 667, 637, 505, 487, 469	**1279**	Roots	16, 38

50	3-*O*-glucosyl-(1→6)-glucosyl-**polygalacic acid** 28-*O*-xylosyl-(1→4)-(2''-*O*-acetyl)rhamnosyl- (1→2)-arabinoside (deapi-2''-*O*-acetylpolygalacin D_3)	1280	1303, 1281, 1149, 1119, 987, 961, 957, 829, 825, 825, 799, 667, 637, 505, 487, 469	**1279**	Roots	16, 17
51	3-*O*-glucosyl-(1→6)-glucosyl-**polygalacic acid** 28-*O*-xylosyl-(1→4)-(3''-*O*-acetyl)rhamnosyl- (1→2)-arabinoside (deapi-3''-*O*-acetylpolygalacin D_3)	1280	1303, 1281, 1149, 1119, 987, 961, 957, 829, 825, 825, 799, 667, 637, 505, 487, 469	**1279**	Roots	16
52	3-*O*-glucosyl-(1→6)-glucosyl-(1→6)-glucosyl-**platycodigenin** 28-*O*-rhamnosyl-(1→2)-arabinoside (platycoside G_2)	1284	1307, **1285**, 1139, 1007, 1123, 977, 961, 845, 815, 683, 653, 521, 503, 485, 467	**1283, 1005, 987, 943, 843, 681, 651, 519**	Roots	14, 20, 24
53	3-*O*-glucosyl-(1→3)-glucosyl-**platycodigenin** 28-*O*-xylosyl-(1→4)-(2''-*O*-acetyl)rhamnosyl-(1→2)-arabinoside (deapi-2''-*O*-acetylplatycodin D_2)	1296	1319, 1297, 1165, 1135, 1003, 977, 973, 845, 841, 815, 683, 653, 521, 503, 485, 467	1295, 1253, 1235, 843, 825, 681, 663, 519	Roots	17, 26
54	3-*O*-glucosyl-(1→3)-glucosyl-**polygalacic acid** 28-*O*-apiosyl-(1→3)-xylosyl-(1→4)-rhamnosyl-(1→2)-arabinoside (polygalacin D_2)	1370	**1393**, 1371, 1239, 1209, 1107, 1077, 1047, 961, 945, 915, 829, 799, 783, 667, 637, 505, 487, 469	**1369, 1351, 1237, 1207, 1105, 827, 665, 647, 503**	Roots	2, 15, 16, 17, 19, 26, 31, 34
55	3-*O*-glucosyl-(1→6)-glucosyl-**polygalacic acid** 28-*O*-apiosyl-(1→3)-xylosyl-(1→4)-rhamnosyl-(1→2)-arabinoside (polygalacin D_3; platycoside G_3)	1370	1393, **1371**, 1239, 1209, 1107, 1077, 1047, 961, 945, 915, 829, 799, 783, 667, 637, 505, 487, 469		Roots	14, 32, 34
56	3-*O*-glucosyl-(1→6)-glucosyl-**platycogenic acid A** lactone 28-*O*-apiosyl-(1→3)-xylosyl-(1→4)-rhamnosyl-(1→2)-arabinoside (platyconic acid B lactone)	1382	**1405**, 1383, 1251, 1221, 1119, 1089, 1059, 973, 957, 927, 841, 811, 795, 649, 517, 499		Roots	2, 34

57	3-*O*-glucosyl-(1→3)-glucosyl-**platycodigenin** 28-*O*-apiosyl-(1→3)-xylosyl-(1→4)-rhamnosyl-(1→2)-arabinoside (platycodin D_2)	1386	1409, **1387**, 1255, 1225, 1123, 1093, 1063, 977, 961, 931, 845, 815, 799, 683, 653, 521, 503, 485, 467	**1385, 1121, 843, 681**	Leaves, Stems, Roots	2, 3, 15, 18, 19, 20, 24, 31, 32, 34
58	3-*O*-glucosyl-(1→6)-glucosyl-**platycodigenin** 28-*O*-apiosyl-(1→3)-xylosyl-(1→4)-rhamnosyl-(1→2)-arabinoside (platycodin D_3)	1386	**1409, 1387, 1255**, 1225, **1123**, 1093, 1063, 977, 961, 931, **845**, 815, 799, 683, 653, 521, 503, 485, 467	**1385, 1253, 1223, 1121, 843, 663**	Leaves, Stems, Roots	3, 11, 12, 13, 14, 16, 17, 18, 19, 20, 23, 24, 31, 32, 34, 37, 38
59	3-*O*-glucosyl-(1→6)-glucosyl-(1→6)-glucosyl-**polygalacic acid** 28-*O*-xylosyl-(1→4)-rhamnosyl-(1→2)-arabinoside (deapi-polygalacin E; platycoside I)	1400	**1423**, 1401, 1269, 1239, 1123, 1107, 1077, 991, 961, 945, 915, 829, 799, 783, 667, 637, 505, 487, 469	**1399, 989**	Roots	8, 16, 24
60	3-*O*-glucosyl-(1→3)-glucosyl-**polygalacic acid** 28-*O*-apiosyl-(1→3)-xylosyl-(1→4)-(2''-*O*-acetyl)-rhamnosyl-(1→2)-arabinoside (2''-*O*-acetylpolygalacin D_2)	1412	1435, **1413**, 1281, 1251, 1149, 1119, 1089, 987, 961, 957, 829, 825, 799, 667, 637, 505, 487, 469	**1411, 1279, 827, 665**	Roots	2, 15, 24, 30, 32, 34
61	3-*O*-glucosyl-(1→3)-glucosyl-**polygalacic acid** 28-*O*-apiosyl-(1→3)-xylosyl-(1→4)-(3''-*O*-acetyl)-rhamnosyl-(1→2)-arabinoside (3''-*O*-acetylpolygalacin D_2)	1412	1435, **1413**, 1281, 1251, 1149, 1119, 1089, 987, 961, 957, 829, 825, 799, 667, 637, 505, 487, 469	**1411, 1279, 827, 647**	Roots	2, 15, 24, 32
62	3-*O*-glucosyl-(1→6)-glucosyl-**polygalacic acid** 28-*O*-apiosyl-(1→3)-xylosyl-(1→4)-(2''-*O*-acetyl)-rhamnosyl-(1→2)-arabinoside (2''-*O*-acetylpolygalacin D_3)	1412	1435, 1413, 1281, 1251, 1149, 1119, 1089, 987, 961, 957, 829, 825, 799, 667, 637, 505, 487, 469	**1411**	Roots	16

63	3-*O*-glucosyl-(1→6)-glucosyl-**polygalacic acid** 28-*O*-apiosyl-(1→3)-xylosyl-(1→4)-(3''-*O*-acetyl)-rhamnosyl-(1→2)-arabinoside (3''-*O*-acetylpolygalacin D_3)	1412	1435, 1413, 1281, 1251, 1149, 1119, 1089, 987, 961, 957, 829, 825, 799, 667, 637, 505, 487, 469	**1411, 1147**	Roots	16, 17
64	3-*O*-glucosyl-(1→6)-glucosyl-(1→6)-glucosyl-**platycodigenin** 28-*O*-xylosyl-(1→4)-rhamnosyl-(1→2)-arabinoside (deapi-platycoside E; platycoside G_1)	1416	**1439, 1417, 1285**, 1255, **1139**, 1123, 1093, **1007**, 977, 961, 931, **845**, 815, 799, **683**, 653, **521**, 503, 485, 467	**1415, 1283, 1253, 1091, 1005, 843 , 681, 519**	Roots	3, 4, 5, 11, 12, 13, 14, 16, 17, 18, 19, 20, 22, 23, 24, 27, 30, 31, 32, 34, 38
65	3-*O*-glucosyl-(1→3)-glucosyl-**platycodigenin** 28-*O*-apiosyl-(1→3)-xylosyl-(1→4)-(2''-*O*-acetyl)-rhamnosyl-(1→2)-arabinoside (2''-*O*-acetylplatycodin D_2; platycodin V)	1428	**1451, 1429,** 1297, 1267, 1165, 1135, 1105, 1003, 977, 973, 845, 841, 815, 683, 653, 521, 503, 485, 467	1427, 1385, 1367, 1295, 843, 681, 681, 519	Roots	15, 24, 26, 32, 34
66	3-*O*-glucosyl-(1→3)-glucosyl-**platycodigenin** 28-*O*-apiosyl-(1→3)-xylosyl-(1→4)-(3''-*O*-acetyl)-rhamnosyl-(1→2)-arabinoside (3''-*O*-acetylplatycodin D_2)	1428	**1451, 1429,** 1297, 1267, 1165, 1135, 1105, 1003, 977, 973, 845, 841, 815, 683, 653, 521, 503, 485, 467	1427, 1385, 1367, 843, 519	Roots	15, 17, 26, 32, 34, 38
67	3-*O*-glucosyl-(1→6)-glucosyl-**platycodigenin** 28-*O*-apiosyl-(1→3)-xylosyl-(1→4)-(2''-*O*-acetyl)-rhamnosyl-(1→2)-arabinoside (2''-*O*-acetylplatycodin D_3)	1428	**1451, 1429,** 1297, 1267, 1165, 1135, 1105, 1003, 977, 973, 845, 841, 815, 683, 653, 521, 503, 485, 467	1427, 1385, 1367, 843, 519	Roots	26, 38
68	3-*O*-glucosyl-(1→6)-glucosyl-**platycodigenin** 28-*O*-apiosyl-(1→3)-xylosyl-(1→4)-(3''-*O*-acetyl)-rhamnosyl-(1→2)-arabinoside (3''-*O*-acetylplatycodin D_3)	1428	1451, 1429, 1297, 1267, 1165, 1135, 1105, 1003, 977, 973, 845, 841, 815, 683, 653, 521, 503, 485, 467	1427, 1385, 1367, 843, 681, 519	Roots	26, 38

69	3-*O*-glucosyl-(1→6)-glucosyl-(1→6)-glucosyl-**polygalacic acid** 28-*O*-apiosyl-(1→3)-xylosyl-(1→4)-rhamnosyl-(1→2)-arabinoside (platycoside D)	1532	1555, 1533, 1401, 1371, 1269, 1239, 1209, 1123, 1107, 1077, 991, 961, 945, 915, 829, 799, 783, 667, 637, 505, 487, 469	1531, 989	Roots	20, 24
70	3-*O*-glucosyl-(1→6)-glucosyl-(1→6)-glucosyl-**platycodigenin** 28-*O*-apiosyl-(1→3)-xylosyl-(1→4)-rhamnosyl-(1→2)-arabinoside (platycoside E)	1548	1571, **1549, 1417**, 1387, **1285**, 1255, 1225, **1139**, 1123, 1093, **1007**, 977, 961, 931, **845**, 815, 799, **683**, 653, **521**, 503, 485, 467	1547, 1415, 1385, 1283, 1223, 1005	Roots	3, 11, 12, 13, 14, 16, 17, 18, 19, 20, 22, 23, 24, 27, 30, 31, 32, 34, 38

1)MW: molecular weight.

2)Bold font indicates reported product ions and the remaining proposed product ions in positive ESI-ionization mode(*m*/*z*)

3)Normal font indicates reported product ions in negative ESI-ionization mode (*m*/*z*, $[M-H]^-$).

4)Previous reports were referred to construct on LC-MS library. (1) Akiyama et al., 1972, (2) Choi et al., 2010, (3) Choi et al., 2008, (4) Fu et al., 2006, (5) Fu et al., 2007, (6) Fu et al., 2011, (7) Fu et al., 2006, (8) Fu et al., 2006, (9) Fu et al., 2006, (10) Ha et al., 2011, (11) Ha et al., 2009, (12) Ha et al., 2010, (13) Ha et al., 2006, (14) He et al., 2005, (15) Ishii et al., 1978, (16) Jeong et al., 2010, (17) Jeong et al., 2014, (18) Kim et al., 2013, (19) Kim et al., 2013, (20) Lee et al., 2015, (21) Li et al., 2012, (22) Li et al., 2007, (23) Li et al., 2010, (24) Ling et al., 2014, (25) Ma et al., 2013, (26) Na et al., 2008, (27) Qian-qian et al., 2014, (28) Qin et al., 2014, (29) Qiu et al., 2018, (30) Ryu et al., 2012, (31) Sun et al., 2011, (32) Wang et al., 2017, (33) Xu et al., 2005, (34) Yoo et al., 2011, (35) Zhan et al., 2012, (36) Zhang et al., 2007, (37) Zhao et al., 2005, (38) Zhao et al., 2018.
